# DNA-influenced automated behavior detection on twitter through relative entropy

**DOI:** 10.1038/s41598-022-11854-w

**Published:** 2022-05-16

**Authors:** Rosario Gilmary, Akila Venkatesan, Govindasamy Vaiyapuri, Deepikashini Balamurali

**Affiliations:** 1 Department of Computer Science and Engineering, Pondicherry Engineering College, Pondicherry, India; 2 Department of Information Technology, Pondicherry Engineering College, Pondicherry, India

**Keywords:** Engineering, Mathematics and computing

## Abstract

Twitter is a renowned microblogging site that allows users to interact using tweets and it has almost reached 206 million daily active users by the second quarter of 2021. The ratio of Twitter bots has risen in tandem with their popularity. Bot detection is critical for combating misinformation and protecting the credibility of online disclosures. Current bot detection approaches rely on the Twitosphere’s topological structure, ignoring the heterogeneity among the profiles. Moreover, most techniques incorporate supervised learning, which depends strongly on large-scale training sets. Therefore, to overcome these issues, we proposed a novel entropy-based framework to detect correlated bots leveraging only user behavior. Specifically, real-time data of users is collected and their online behaviors are modeled as DNA sequences. We then determine the probability distribution of DNA sequences and compute relative entropy to evaluate the distance between the distributions. Accounts with entropy values less than a fixed threshold represent bots. Extensive experiments conducted in real-time Twitter data prove that the proposed detection technique outperforms state-of-the-art approaches with precision = 0.9471, recall = 0.9682, F1 score = 0.9511, and accuracy = 0.9457.

## Introduction

Twitter is a popular microblogging platform that allows users to express their opinions and form social connections. Because of characteristics like an open platform and anonymity, it has become an ideal medium for the growth of bots^1^. Twitter bots are software applications that run automated tasks. Although there is a common misconception that all bots are malicious, Twitter's guidelines permit the use of automated bots. However, it forbids the use of bots for illegal purposes^2^. Some bots such as @big ben clock are benign, which mimics the original Big Ben clock^3^. There are also other malicious bots that engage in various illegal activities such as spamming, generating fake popularity, publishing misinformation, online harassment, terrorism, and restricting free speech rights^4^. One of the recent issues with bots is the spread of misinformation regarding the COVID-19 pandemic. According to an analysis on a known bot dataset, nearly 66% of profiles spreading misinformation on COVID-19 are bots^5^. They were disseminating conspiracy theories like QAnon and spreading URLs from partisan news sites^6^. A real-life consequence of such misinformation includes inadequate hydroxychloroquine drug because of strong demand from people who believe it will build protection against COVID-19^7^. Also, misleading information has a negative effect on people’s intentions to get vaccinated against COVID-19^8^. It is also proven that Twitter bots have played a crucial part in different scenarios like public elections^9^ and stock microblogs^10^. Therefore, it becomes essential to remove malicious bots from the Twitter environment. Most of the bot detection approaches analyze multiple features and incorporate machine learning classifiers trained with known bot datasets to determine whether the profile is automated or not^11^. However, feature selection is a challenging task while using machine learning classifiers^12^.

Feature-like user behaviors are modeled and analyzed for different objectives. A contemporary line of research has detected bots by analyzing user behaviors using bioinformatics approaches^13–17^. In this research, we proposed a novel approach to detect correlated bots leveraging only user behaviors. A DNA base (A, C, T, or G) is used to define the online user activity performed. Thus, the string of DNA corresponds to the sequence of activities in the user's timeline. The DNA sequences are expressed as probability distributions and, their similarity degrees are quantified using relative entropy. Here, the degree of similarity present in the probability distributions acts as an indicator of automation. Entropy ranges between 0 and 1, where 0 signifies that the distributions have similar information^18^. Thus, as the entropy decreases, the probability of the corresponding profile being a bot increases.

The following are the primary contributions of the proposed work.

- The proposed approach analyses the user behaviors by considering the profile’s timeline and characterizes them as DNA sequences.
- We compute relative entropy on the probability distributions corresponding to the DNA sequences and it estimates the degree of similarity present. Bots are classified from humans by evaluating the entropy scores.
- The performance of the proposed approach is computed in the real-time Twitter dataset and compared with the state-of-the-art techniques.

This paper is structured as follows. Section 2, discusses the literature survey in brief. Section 3 presents the proposed entropy-based automation detection on Twitter using DNA modeling. Section 4 describes the experimental design and discussions and highlights an overview of selection of decision threshold, empirical outcomes of the proposed model, and comparison with state-of-the-art approaches. It also explains the real-world Twitter dataset collection and baseline dataset considered for performance evaluation. Section 5 concludes the paper.

## Related work

The literature presents the research that has achieved intriguing outcomes related to our proposal. Related works are discussed under two broad categories. The first deals with the entropy-based methods. The second reviews DNA modeling-based approaches for bot detection.

### Entropy based methods

Multiple research works have paid attention to entropy-based features to detect automated behavior on Twitter. Inspired by them, a bot detection approach using the approximate entropy and sample entropy has been proposed^19^. The number of tweets posted periodically by a user is the primary temporal feature considered. The amount of regularity present in the data is quantified using an entropy estimate which functions as an indicator of the bot. Experiments on real-time datasets show that approximate entropy and sample entropy have provided significant outcomes of 85% accuracy and 80% accuracy, considering only a single feature. The significance of entropy in bot detection is proven by the strong negative correlation between entropy and class of profile (bot or human), using point-biserial correlation.

Chu et al.^20^ analyzed features like tweeting behavior, tweet content, user features and classified them as a bot, cyborg, or human. The modal uses entropy estimate and a bot detection element. The entropy is computed on the time-based feature, and the bot detection component employs a Bayesian classification to examine tweet content. Further, the random forest method classifies the account as bot or human. Their results showed that the entropy achieved the highest discriminating score among the features investigated, with an accuracy of 82.8%. Besides, the model achieved a 96% True Positive Rate.

Gia et al.^21,22^ used entropy in supervised machine learning classifiers to detect chatbots from human accounts. The modal consists of two elements: an entropy-based classifier and a machine learning classifier. The entropy-based classifier examines the time between messages and its size to evaluate the complexity of the chat flow. Whereas, the Bayesian machine learning classifier analyses the content of the messages. The evaluation is conducted based on both supervised training and entropy classifier-based training. This model achieved 99% True Positive Rate.

Goesh et al.^23^ emphasized retweeting dynamics and embedded URLs to detect bots. The model computed the entropies of the time interval distribution and user distribution in retweeted URLs. The time-interval entropy increases as the time intervals between two consecutive retweets differ. Similarly, the user entropy increases if every user retweets a particular tweet only once. In addition, the model uses a Support Vector Machine classifier for training and achieved an F measure of 61% in performance evaluation.

Entropy is used in disaster-based event detection where the technique involves computing hashtag entropy, time interval entropy, user entropy, and location entropy from tweets and retweets^24^. The automation is detected by exploiting the profile’s retweeting activities with time interval entropy as human accounts have different inter-arrival rates. This indicates that they are likely to be equally distributed. Contrarily, the frequency of retweeting bots showed significant distributions as they retweeted at regular time intervals.

Perdana et al.^25^ introduced an unsupervised entropy-based bot detection technique that uses time interval entropy and tweet similarity as the key features. The Uni-gram matching method of similarity computes the similarity in tweets. The final score that classifies bots from humans is determined from the aggregation of the time interval entropy and tweet similarity measure with their weighting factor. The proposed modal yields a True Positive Rate of 94.74%.

### DNA modeling based methods

Inspired by genetics, previous studies^13–17^ have modeled the behavioral activities of Twitter users using DNA sequences that were generated from the tweets posted by the user accounts. The metric for detecting automation in the profiles is sequence similarity. The similarity among the DNA sequences is evaluated using the Longest Common Substring (LCS). Analyzing the LCS curves developed from the type and content of tweets implies that modeling based on the type provides more efficiency^13,14^. DNA modeling is integrated with genetic algorithms to create evolved DNA sequences of new bots^15^. Mutation and crossover are the genetic algorithms employed to develop modern bots. The evolved bot behaviors tested by the advanced bot detection system prove that they succeed in evading the detection. In addition, the research examines the distributions in human behaviors, which are proven to be intensely heterogeneous^16,17^.

### Inference

Previous studies by Chu et al.^20^ and Gilmary et al.^19^ proved that entropy accurately reflects the difference between bot and human behaviors. Although entropy estimate is significant in bot detection, there are not many studies on it. The existing entropy-based supervised approaches addressed in the literature have many shortcomings. These techniques employ a broad range of features wherein extracting certain features from Twitter is time-consuming and expensive^12^. A labelled dataset that includes the entropy-based properties and behavior of a diverse population of bots^26^ is required to train supervised machine learning algorithms. It is difficult to detect generic bots with a limited training set that has a specific type of bots like fake followers or social bots. Furthermore, bots evolve^15^, machine learning classifiers learned with outdated data fail to detect evolved bots^27^. Besides, these data do not reflect the current features of bots, which are the result of updated Twitter policy 2020^28^. The drawbacks of supervised bot detection strategies can be improved by using semi-supervised approaches.

Bot detection based on DNA modeling is a relatively new field of study. It is sufficiently versatile to identify bot behaviors without relying on specific attributes. Thus there are more opportunities for improvement. LCS is presently being used to recognize bots, and it only detects a group of bots that follow the same pattern. Hence, bots that follow unique patterns go undetected.

The proposed technique addresses the shortcomings of the literature. Based on the DNA profiling paradigm, we extract DNA sequences that characterize the user's timeline. We then detect the correlated bots from the similarity index computed using the relative entropy in DNA sequences. Through this technique, correlated bots that follow different patterns are accurately detected by using only a single feature.

The main advantages of this research are only a single feature: the account timeline is used. Further, the modal does not use any traditional supervised classifiers. Thus, there is no requirement for the training phase. Implementation through a semi-supervised approach lessens the requirement of the manually labelled dataset. Thus, annotated data used in the experiments are reduced. With the usage of minimal resources, the proposed approach detects generic correlated bots rather than any particular type of bots as in supervised techniques.

## Proposed work

**Figure 1:**
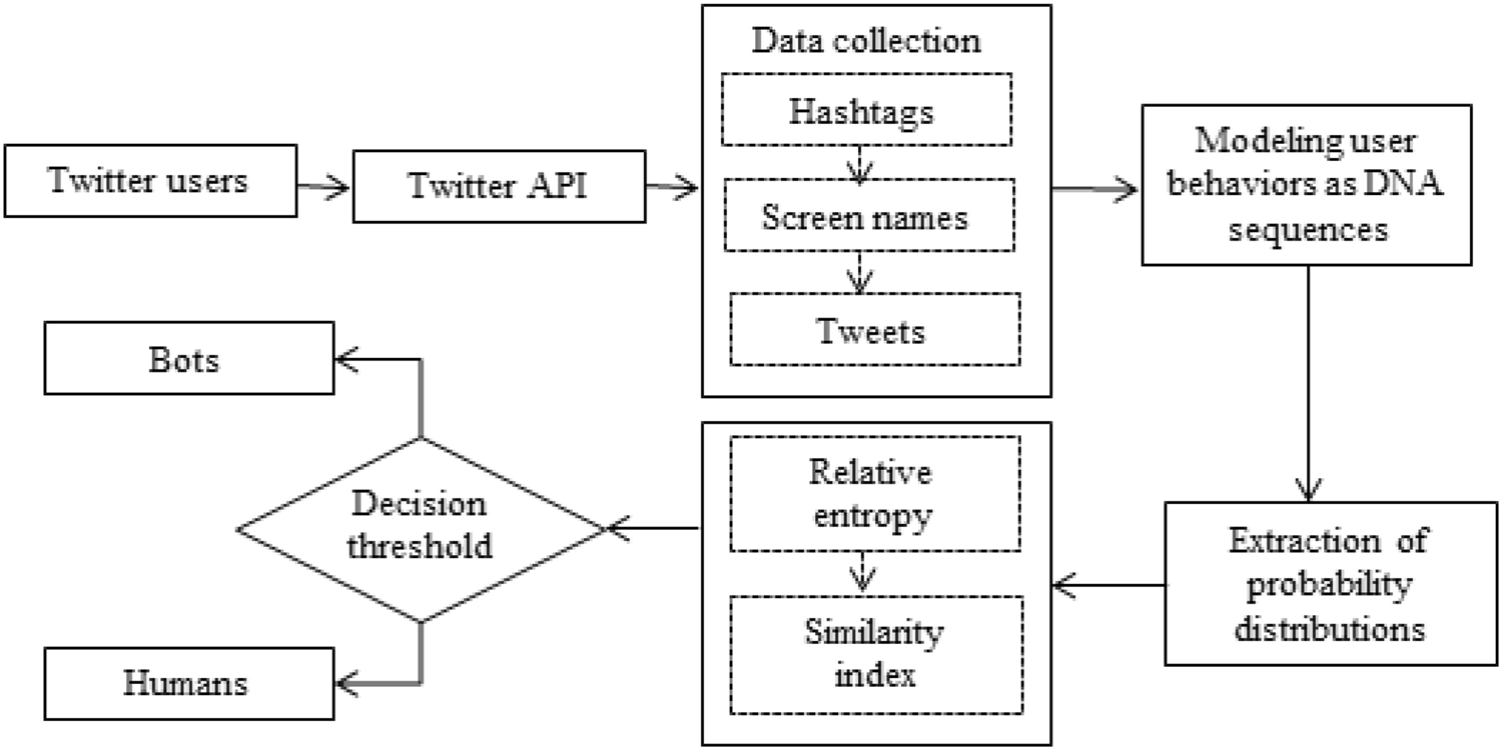
Experimental design of proposed work.

In our previous work, we calculate entropy on the temporal feature of user accounts to detect bots through auto-correlation^19^. In this paper, we extend our previous work to detect correlated bots by computing relative entropy on user behaviors. Figure 1 explains the framework of the proposed bot detection approach. The designed approach includes the collection of real-time datasets followed by three main phases. In the initial phase, we model the user behaviors as DNA sequences, as explained in Sect. 3.1. Section 3.2 gives a detailed overview of constructing the corresponding probability distributions. Finally, in Sect. 3.3, we use relative entropy to analyze the similarity, which acts as a parameter to detect bots.

### Modeling user behavior as DNA sequence

The user behaviors are modeled as DNA sequences by assigning a DNA base to each activity performed by a user. Thus, the DNA sequence signifies the user’s timeline. The number and interpretations of the DNA bases can be modified based on the requirement. We define a user profile (U) as a string of DNA bases,

1 $$ U = \left\{ {b_{1} , b_{2} , \ldots ,b_{n} } \right\} b_{i} \in F \forall i = 1,2, \ldots n $$

where, the DNA bases $\left( {b_{i} } \right)$ in $U$ are the elements from the finite set $F.$

2 $$ F = \left\{ {F_{1} ,F_{2} , \ldots ,F_{N} } \right\} F_{i} \ne F_{j} \forall i,j = 1,2, \ldots ,N i \ne j $$

Each user activity is encoded by assigning an ${F}_{i}$ element. We obtain the user's DNA sequence by scanning their timeline chronologically and assigning appropriate DNA bases. In the proposed method, we assign DNA bases based on the types and content of tweets shared. Since, these features are proved effective in detecting bots^13,14,29,30^, each tweet posted by a user is assigned a unique DNA base as presented in Table 1 (i.e.) A-plain tweet, T- plain mention, G- plain retweet, C- tweet with media/URLs). For each profile, we can extract a DNA sequence of length 3200 tweets as the Twitter API limits 3200 tweets.

**Table 1:** Labelling and descriptions of DNA base in user profile.

Base	Description
*b*_*1*_	A ← plain tweet
*b*_*2*_	T ← plain mention
*b*_*3*_	G ← plain retweet
*b*_*4*_	C ← tweet with media / URLs

### Probability distribution of DNA sequence

Initially, we assign four vector values corresponding to the four bases between 0 and 1 to obtain probability distributions. The values are assigned in accordance with the significance of a particular DNA base in bot detection. In this paradigm, we have assigned $\overrightarrow{T}=0.2,\overrightarrow{A}=0.4,\overrightarrow{G}=0.6, and \overrightarrow{C}=0.8.$ Larger vector values are given to the DNA base representing retweets and tweets with media/URLs because most bots spread retweets/media/URLs. Then the DNA sequences are expressed as discrete probability distributions ^31^.

We define the probability distribution of the DNA sequence of length *n* as (${p}_{1},{p}_{2},{p}_{3},\dots ,{p}_{n})$,

3 $$ p_{i} = \frac{{\alpha_{i} - \vec{\beta }_{i} }}{{\frac{1}{2}n\left( {n + 1} \right) - \beta_{n} }} $$

where ${(\alpha }_{i},{\beta }_{i})$ represents the position of ${i}^{th}$ base in DNA sequence and ${\overrightarrow{\beta }}_{i}$ represents the vector value of the corresponding ${i}^{th}$ base.${\beta }_{n}$ is calculated by summing the vectors which represent the bases in the DNA sequence. For example, the probability distribution of the DNA sequence (ATGC) is,

$$ A:\vec{\beta }_{1} = 0.4,T: \vec{\beta }_{2} = 0.2, G:\vec{\beta }_{3} = 0.6,C:\vec{\beta }_{4} = 0.8, and \beta_{n} = \left( {0.4 + 0.2 + 0.6 + 0.8 = 2} \right) $$

$$ (p_{1} ,p_{2} ,p_{3} ,p_{4} ) = \left( {\frac{1 - 0.4}{{\left( {{\raise0.7ex\hbox{$1$} \!\mathord{\left/ {\vphantom {1 2}}\right.\kern-\nulldelimiterspace} \!\lower0.7ex\hbox{$2$}}.4.5} \right) - 2}},\frac{2 - 0.2}{{\left( {{\raise0.7ex\hbox{$1$} \!\mathord{\left/ {\vphantom {1 2}}\right.\kern-\nulldelimiterspace} \!\lower0.7ex\hbox{$2$}}.4.5} \right) - 2}},\frac{3 - 0.6}{{\left( {{\raise0.7ex\hbox{$1$} \!\mathord{\left/ {\vphantom {1 2}}\right.\kern-\nulldelimiterspace} \!\lower0.7ex\hbox{$2$}}.4.5} \right) - 2}},\frac{4 - 0.8}{{\left( {{\raise0.7ex\hbox{$1$} \!\mathord{\left/ {\vphantom {1 2}}\right.\kern-\nulldelimiterspace} \!\lower0.7ex\hbox{$2$}}.4.5} \right) - 2}}} \right) $$

$$ = \left( {0.0750,0.2250,0.3000,0.4000} \right). $$

Proof of discrete probability distribution:

1. $$\sum_{i=1}^{n}{p}_{i}=\sum_{i=1}^{n}\frac{{\alpha }_{i}-{\overrightarrow{\beta }}_{i}}{\frac{1}{2}n\left(n+1\right)-{\beta }_{n}}=\frac{\sum_{i=1}^{n}{\alpha }_{i}-{\sum }_{i=1}^{n}{\overrightarrow{\beta }}_{i}}{\frac{1}{2}n\left(n+1\right)-{\beta }_{n}}$$
  $$=\frac{\frac{1}{2}n\left(n+1\right)-{\beta }_{n}}{\frac{1}{2}n\left(n+1\right)-{\beta }_{n}}=1.$$
2. Since $$0<{\overrightarrow{\beta }}_{i}<1 and 1\le {\alpha }_{i}\le n,$$ $${\alpha }_{i}-{\overrightarrow{\beta }}_{i}\le {\alpha }_{i}\le n.$$
  $${\beta }_{n}=\sum_{i=1}^{n}{\overrightarrow{\beta }}_{i}<n, so \frac{1}{2}n\left(n+1\right)-{\beta }_{n}>\frac{1}{2}n\left(n+1\right)-n.$$

Thus,

$$ p_{i}  = \frac{{\alpha _{i}  - \vec{\beta }_{i} }}{{\frac{1}{2}n\left( {n + 1} \right) - \beta _{n} }}{ < }\frac{n}{{\frac{1}{2}n\left( {n + 1} \right) - n}} = \frac{1}{{\left( {n + 1/2} \right) - 1}} = \frac{2}{{n - 1}} $$

$$\mathrm{ So},\mathrm{ if }n\ge 3, {p}_{i}<1.$$

$${\alpha }_{i}-{\overrightarrow{\beta }}_{i}>0, and \frac{1}{2}n\left(n+1\right)-{\beta }_{n}>\frac{1}{2}n\left(n+1\right)-n=\frac{n\left(n-1\right)}{2}>0\, when\, n\ge 3.$$

$$\mathrm{So}, {p}_{i}>0.$$

Therefore, if $$n\ge 3, 0<{p}_{i}<1.$$

From (1) and (2), we can prove (${p}_{1},{p}_{2},{p}_{3},\dots ,{p}_{n})$ is a discrete probability distribution.

### Similarity measure by relative entropy

Entropy is a metric that measures the degree of randomness in a dataset^32^. In DNA, entropy quantifies the repeatability in the sequences^33^. We compute the probability distributions of all DNA sequences corresponding to individual user profiles. Finally, we estimate the relative entropy^34,35^ between all pairs of distributions to determine the similarity index. In this research, we use the similarity measure as an indicator to identify bots. All entropy measures have the same property where complete random data gets the highest entropy score. And a low entropy score indicates the sequence comprising of repeating patterns. Thus, if the entropy of a pair of distributions is low, the probability of the corresponding profiles being correlated bots is high.

Problem definition: Given the discrete probability distributions ${\upmu }_{1}=({p}_{1},{p}_{2},\dots ,{p}_{n})$ and ${\upmu }_{2}=({q}_{1},{q}_{2},\dots ,{q}_{n})$ on a universe $X$ for a pair of DNA sequences,

The relative entropy ${R}_{en}\left({\upmu }_{1},{\upmu }_{2}\right)$ of ${\upmu }_{1}$ with respect to ${\upmu }_{2}$ is defined as follows,

4 $$ R_{en} \left( {_{1} ,_{2} } \right) = \mathop \sum \limits_{x \in X} p_{1} \left( x \right)log\frac{{p_{1} \left( x \right)}}{{q_{2} \left( x \right)}} $$

The relative entropy ${R}_{en}\left({\upmu }_{2},{\upmu }_{1}\right)$ of ${\upmu }_{2}$ with respect to ${\upmu }_{1}$ is defined as follows,

5 $$ R_{en} \left( {_{2} ,_{1} } \right) = \mathop \sum \limits_{x \in X} q_{1} \left( x \right)log\frac{{q_{1} \left( x \right)}}{{p_{2} \left( x \right)}} $$

The similarity index is defined as follows,

6 $$ d(_{1} ,_{2} ) = \frac{{R_{en} \left( {_{1} ,_{2} } \right) + R_{en} \left( {_{2} ,_{1} } \right)}}{2} $$

Thus, we can compute the similarity index between a pair of DNA sequences. Based on the $d({\upmu }_{1},{\upmu }_{2}$) score, the pair of user accounts corresponding to the probability distributions are classified as either a bot or human. The algorithm for computing relative entropy and similarity index is discussed in Algorithm 1.
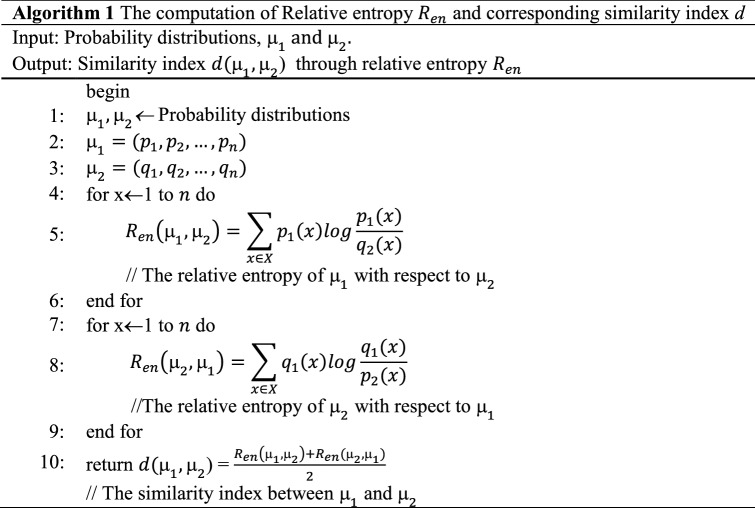


## Experimental design and discussions

This section discusses the experimental setup of the proposed work. As presented in Fig. 1**,** the proposed bot detection approach consists of four components: data collection and annotation, modeling user behaviors as DNA sequences, constructing probability distribution of every DNA sequence, and computation of relative entropy.

### Data collection and annotation

**Figure 2:**
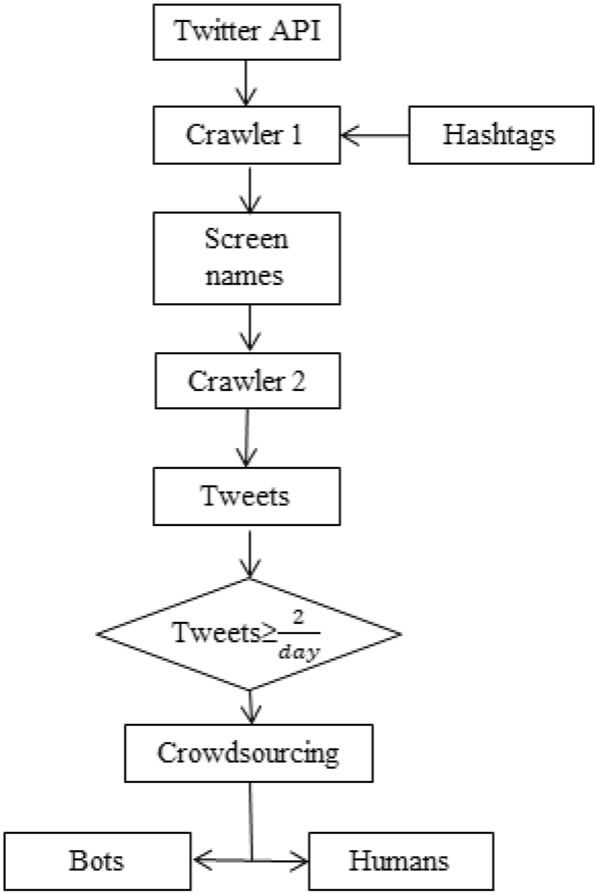
Flow diagram of data collection.

For a plethora of reasons, the proposed research makes use of a new real-world Twitter dataset. The primary reason being that the research centres on Indian bots. Thus, we collected bots from India's most popular hashtags. Secondly, academics acknowledged that there are limited human-labelled Twitter datasets for bot detection^36^. Previous research used bot datasets with certain bot types, such as social bots^27^ or fake followers^12^. For efficient bot detection, the training dataset should reflect the behavior of a broad range of bots rather than a single type. Furthermore, datasets collected with the Twitter API must comply with the latest developer policies^28^. Finally, Twitter deactivates millions of bots every month. As a result, several accounts of old datasets are banned, deleted, or made private^37^. Figure 2 explains the flow chart of data collection through the Twitter API. Since most bots target trending stories, hashtag selection is critical. In this study, the hashtags considered are #corona vaccine, #FarmBills2020, #Indian stock market, #jallikattu, #nepotism, #NRC, #Rights, #sterlite, #Tamil, #Tamil Nadu, #Against Modi, #Farmers protest, and #Narendramodi. These hashtags were active at different periods assuring that the analyses conducted are not biased. A Twitter crawler collects the screen names of profiles that tweet on particular hashtags using the *Standard Search API*. Then, the *user_timeline REST API* extracts datasets of individual profiles by examining the indexed keywords and delivers twitter posts that match the search criteria. The dataset (≈7,353,600 tweets) was extracted between August 2020 and July 2021 in English employing the Twitter Standard API language parameter: *lang* = *“es”*. Using *statuses_count* and *created _at* API attributes, profiles that share at least 2-tweets/day are filtered as research stated that genuine profiles share between 2 and 500 tweets/day^38^.

We build the ground truth of the data collected through crowdsourcing^39^ which labels an account as a bot or human. Crowdsourcing is conducted by a group of Computer Science postgraduates as testers who manually annotate each profile. Each tester inspects 80 profiles and segregates them as a bot or human based on the timeline, account features, photo albums, and profile photos. The group is divided into four teams, and all four teams analyse each profile to increase the classification accuracy. The outcomes of the four teams are aggregated, and the mode of the aggregation is the resultant classification. Twitter also has bots that pose no threat^40^, such profiles are excluded using the Twitter API *Is-Verified* feature. The final dataset comprises ≈2300 profiles of 1094 bots and 1204 humans, each with the following fields: *Tweet-Id*, *Timestamp*, and *Tweet*.

### Baseline dataset

The baseline dataset contains 800 profiles as training data and 1500 profiles as testing data from the complete dataset. We consider two limited datasets: Group_1 and Group_2, of size 400 each, balanced with bots and humans as training datasets. They are used for fixing decision thresholds. We validate the empirical results of the proposed modal in the test datasets. Using the Bootstrap technique, we extract 5 test datasets from 1500 profiles: Test_1, Test_2, Test_3, Test_4, and Test_5 of size 600 each, with 36.8% new profiles^41^. The baseline dataset includes 800 accounts balanced with bots and humans for analyzing DNA patterns and 1200 accounts as testing data from the original data collected. We evaluate the empirical results of the proposed modal in the test datasets. We extract 5 test datasets from 1200 profiles Test_1, Test_2, Test_3, Test_4, and Test_5 using Bootstrap technique^41^.

### Extraction of DNA sequences and probability distributions

In this phase, the DNA sequence corresponding to each Twitter user is extracted, a string encoding the user’s timeline. Each activity performed by the user is encoded with a unique DNA base (i.e.) A-plain tweet, T-plain mention, G-plain retweet, C-tweet with media (photos and URLs). Finally, we define the probability distributions of each DNA sequence as discussed in Sect. 3.2.

### Fixing decision threshold for relative entropy

Twitter bot detection is a binary classification, where the *decision threshold* dichotomizes the profile as either a class bot or class human. Here, the decision threshold is a $d({\upmu }_{1},{\upmu }_{2}$) value in the range between 0 and 1.

- $$d({\upmu }_{1},{\upmu }_{2}) \le \,decision \,threshold=class\, bot$$
- $$d({\upmu }_{1},{\upmu }_{2}) >decsion\, threshold=class \,human$$

The analyses have been conducted in three dimensions: (bots, bots), (bots, humans), and (humans, humans). Table 2 explains the experiments conducted on Group_1 and Group_2 to determine the decision threshold. In each dataset, we perform four iterations with varying number of accounts as shown in Table 2. In every iteration, we calculate $d({\upmu }_{1},{\upmu }_{2}$) for all pairs of combinations within bot set (bots, bots), human set (humans, humans), and bot and human set (bots, humans). We then consider their means as final outcomes. The average $d ({\upmu }_{1},{\upmu }_{2}$) score of (bots, bots) is significantly lesser than (bots, humans) and (humans, humans). This certainly proves that bots are correlated and exhibit similar behavioral patterns. Moreover, the average $d ({\upmu }_{1},{\upmu }_{2}$) scores of (bots, humans) and (humans, humans) are relatively higher because of their heterogeneous patterns. This variation proves that similarity index computed through relative entropy shares a significant relationship with class of Twitter account and, entropy is negatively correlated with bots.

**Table 2:** Analysis conducted to set decision threshold in Group_1 and Group_2.

Datasets	No. of accounts	*d* (Bots, Bots)	*d* (Bots, Humans)	*d* (Humans , Humans)
Group_1	50	0.0093	0.3501	0.4011
	100	0.0282	0.4209	0.4591
	150	0.0829	0.4526	0.4973
	200	0.1054	0.4803	0.5291
Group_2	50	0.0157	0.3690	0.4179
	100	0.0328	0.4259	0.4358
	150	0.0978	0.4531	0.4939
	200	0.1191	0.4950	0.4969
Mean	0.0614	0.4308	0.4663	
Inference		*d*(Bots, Bots) < *d*(Bots, Humans) < *d*(Humans, Humans)		
Decision threshold		Sample Maxima *d* (Bots, Bots) = 0.12		

The strong candidate splitting point of classification is the threshold value that detects all correlated bots (i.e.) (bots, bots). The optimal decision threshold is determined considering the sample maxima of $d ({\upmu }_{1},{\upmu }_{2}$) from all iterations of (bots, bots). As a result, correlated bots that follow multiple patterns can be detected. Observing the readings from Table 2, the optimal decision threshold obtained was $0.12$.

### Performance evaluation 

The performance of the proposed method is analyzed based on the metrics: Precision, Recall, Miss Rate, Accuracy (ACC), F1 score (harmonic mean of recall and precision), and Matthews Correlation Coefficient (MCC). Table 3 illustrates the performance of the proposed technique on the following test datasets: Test_1, Test_2, Test_3, Test_4, and Test_5. Our technique is compared with the entropy-based approach on temporal patterns^19^, the DNA modeling-based research^13,14,16^, and the bot detection tool, Botometer^42,43^.

**Table 3:** Comparison of performance calculated by different techniques on test datasets.

Dataset	Size	Technique	Evaluation metrics					
			Precision	Recall	Miss Rate	Accuracy	F1	MCC
Test_1	600	DNA influenced relative entropy	0.9416	0.9784	0.0216	0.9457	0.9443	0.9010
		Approximate entropy^19^	0.7686	0.9617	0.0383	0.8483	0.8679	0.7295
		Sample entropy^19^	0.7028	0.9626	0.0374	0.7926	0.8243	0.6332
		DNA fingerprinting^13,14^	0.9298	0.7350	0.2650	0.9230	0.9229	0.8470
		Botometer^42,43^	0.6291	0.2911	0.7089	0.4898	0.3690	0.2038
Test_2	600	DNA influenced relative entropy	0.9403	0.9733	0.0267	0.9379	0.9453	0.9042
		Approximate entropy^19^	0.7704	0.9621	0.0379	0.8500	0.8692	0.7324
		Sample entropy^19^	0.7044	0.9633	0.0367	0.7963	0.8256	0.6363
		DNA fingerprinting^13,14^	0.9301	0.8018	0.1982	0.9201	0.9198	0.8590
		Botometer^42,43^	0.6701	0.2967	0.7033	0.4902	0.3897	0.2103
Test_3	600	DNA influenced relative entropy	0.9431	0.9671	0.0329	0.9412	0.9531	0.9137
		Approximate entropy^19^	0.7773	0.9614	0.0386	0.8558	0.8737	0.7422
		Sample entropy^19^	0.7125	0.9625	0.0375	0.7997	0.8313	0.6492
		DNA fingerprinting^13,14^	0.9249	0.7834	0.2166	0.9215	0.9214	0.8530
		Botometer^42,43^	0.6770	0.3048	0.6952	0.5001	0.4089	0.2175
Test_4	600	DNA influenced relative entropy	0.9526	0.9629	0.0371	0.9509	0.9550	0.9200
		Approximate entropy^19^	0.7859	0.9610	0.0390	0.8599	0.8792	0.7473
		Sample entropy^19^	0.7249	0.9623	0.0377	0.8089	0.8397	0.6585
		DNA fingerprinting^13,14^	0.9290	0.7991	0.2009	0.9198	0.9191	0.8390
		Botometer^42,43^	0.6842	0.3057	0.6943	0.5760	0.4190	0.2298
Test_5	600	DNA influenced relative entropy	0.9581	0.9591	0.0409	0.9528	0.9579	0.9273
		Approximate entropy^19^	0.8013	0.9501	0.0499	0.8665	0.8896	0.7548
		Sample entropy^19^	0.7459	0.9513	0.0487	0.8190	0.8537	0.6682
		DNA fingerprinting^13,14^	0.9339	0.8023	0.1977	0.9211	0.9208	0.8495
		Botometer^42,43^	0.6949	0.3091	0.6909	0.5832	0.4281	0.2349

The proposed approach is compared with our previous work that emphasizes on the computation of approximate entropy and sample entropy in temporal patterns. The technique involves autocorrelation analyses and considers only a single feature. Here, individual bots are detected by analyzing the amount of regularity present in the temporal patterns. Further, the relationship between bot accounts and entropy is proven using point-biserial correlation. We examined the performance of approximate entropy and sample entropy in the test_datasets. The outcomes illustrate that approximate entropy detects bots better than sample entropy with the F1 measure = 0.8759 and accuracy = 0.8561. While, Sample entropy produces F1 measure = 0.8349 and accuracy = 0.8033.

We also compared our research with the DNA modeling-based approach. Social Fingerprint^13,14,16^ is the primary concept used in DNA modeling-based research. In their base study, the user activities are characterized as DNA sequences considering three features: tweet, retweet and reply. Lastly, Twitter bots are identified by analysing the similarity in the sequences using the Longest Common Substring (LCS) algorithm.

Lastly, we compared our model with Botometer^42,43^, which was used in various studies^44–46^ as a key feature of their analysis. Thus, it is reasonable to conclude that Botometer is a de-facto bot detection paradigm. It calculates a probability value between 0 and 1 by evaluating 1000 features. The classification accuracy for various thresholds is computed on the datasets Group_1 and Group_2, and the threshold with the best accuracy is considered ideal. According to the empirical findings, we selected threshold = 0.43, which is in line with the Botometer team.

**Figure 3:**
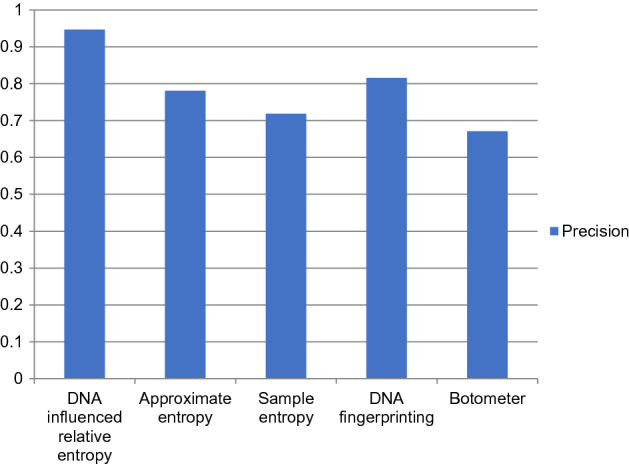
Comparison of Precision metric for different state-of-art approaches.

**Figure 4:**
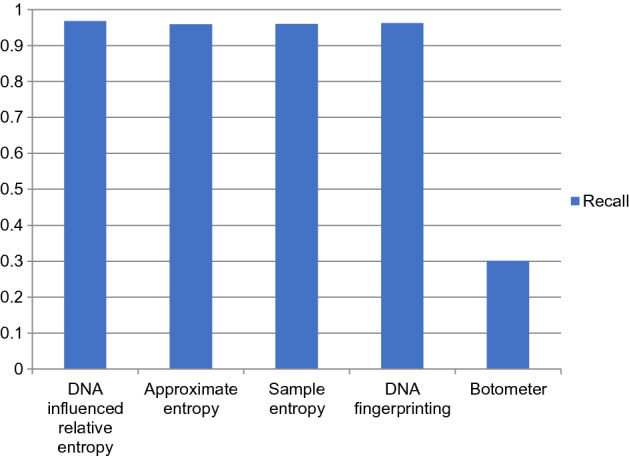
Comparison of Recall metric for different state-of-art approaches.

**Figure 5:**
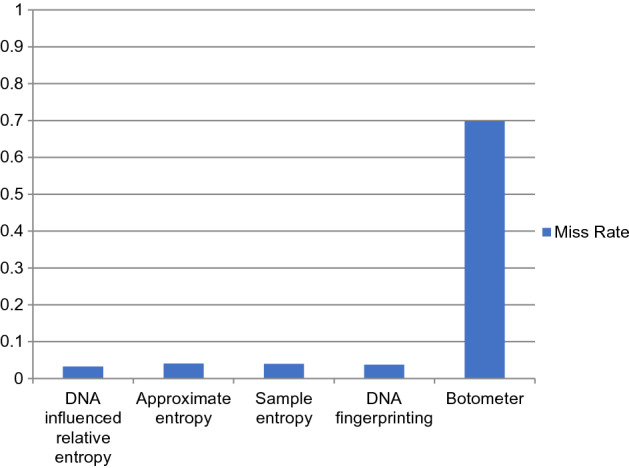
Comparison of Miss rate metric for different state-of-art approaches.

**Figure 6:**
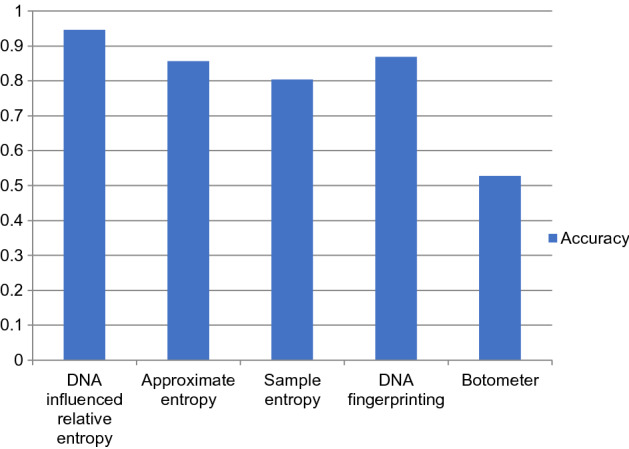
Comparison of Accuracy metric for different state-of-art approaches.

**Figure 7:**
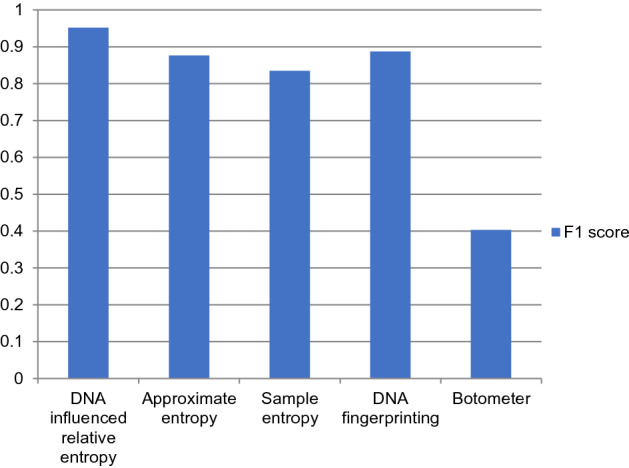
Comparison of F1 score metric for different state-of-art approaches.

**Figure 8:**
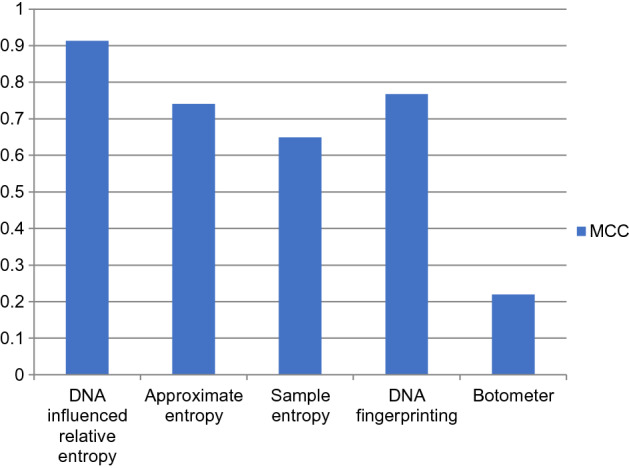
Comparison of MCC metric for different state-of-art approaches.

The performance comparison of the proposed DNA-influenced bot detection using relative entropy and other state-of-the-art approaches are shown in Figs. 3, 4, 5, 6, 7 and 8 for various metrics. The proposed modal outperforms other techniques by achieving an average $F1 score=0.9511$ and average $accuracy=0.9457$. It surpasses the Botometer tool^18,19^ by employing only a single feature (i.e.) profile’s timeline. Social Fingerprinting^13,14,16^ uses LCS which results in detecting only the bots that follow identical patterns. The $recall=0.9681$ achieved by the proposed approach confirms our claim that even correlated bots that follow unique patterns are detected. Also, our technique does not analyze extensive features or a training phase to give higher performance.

Alternatively, we use only the profiles’ timeline. Based on the interesting results, the potency of the entropy-based approach to be deployed in advanced bot detection is foreseen. Relating different entropy modals with compression statistics on user behaviors modeled as DNA sequences is a promising research direction to detect correlated bots.

## Conclusion

In this research, a novel bot detection framework has been designed by using only a single feature: the user's timeline. The experiments were conducted in real-time Twitter datasets collected through updated Twitter API with 2020 Twitter developer policies. The dataset includes 1094 bots and 1204 humans, each with the following fields: *Tweet-Id*, *Timestamp*, and *Tweet*. The study focuses only on the tweet posted on the user’s timeline. For every Twitter profile, their DNA sequence is extracted with four bases A (plain tweet), T (plain mention), G (plain retweet), and C (tweet with media/URLs), and expressed them as probability distributions. Lastly, we compute the similarity index $d({\upmu }_{1},{\upmu }_{2}$) from the mean of relative entropies, ${R}_{en}\left({\upmu }_{1},{\upmu }_{2}\right)$ and ${R}_{en}\left({\upmu }_{2},{\upmu }_{1}\right)$ for all pairs of probability distributions to detect correlated bots. The bottom line of our proposed research is to determine the similarity degree between probability distributions, which serves as an indicator for bot detection. The Twitter profile under examination is classified as a bot or legitimate profile based on the similarity score derived from relative entropies. In a nutshell, correlated bots have higher similarities, resulting in low entropy. The resultant performance metric scores are the average of outcomes of test_datasets. We have compared the performance of DNA-influenced automated behavior detection on Twitter through Relative entropy with the bot detection tool, Botometer^42,43^ and DNA fingerprinting^13,14^. Our technique provided significant results than state-of-the-art approaches with F1 measure = 0.9511 and accuracy = 0.9457.

The merits of this research work are multifold. The proposed DNA-influenced automated behavior detection on Twitter through Relative entropy detects Twitter bots with better accuracy, F1 score, and recall rate. It has enhanced performance by identifying generic bots rather than any specific type. The proposed modal leverages only one primary feature: user timeline. It downsizes the amount of annotated data used. Since the modal does not use any typical machine learning algorithms, it does not have any training phase. Thus, the proposed technique detects correlated bots with minimal resources.

For future research, we plan to extend the DNA-based modeling with the temporal dimension of the tweeting activity. Both tine-based features and timeline features can be considered together to detect correlated bots that are active at particular time periods. The temporal features and timeline features function complementary to each other to design a more robust bot detection paradigm. A novel model that uses combination of Tweet rates with different sampling periods and timeline activities with entropy estimate is a promising research direction.
